# Sex and formula supplementation are correlates of fecal calprotectin in premature infants at 10–18 months corrected age

**DOI:** 10.3389/fped.2026.1743276

**Published:** 2026-10-14

**Authors:** Yaqi Liu, Yi Guo, Xiaoting Lu, Beiquan Li, Wenjing Chang, Kan Ye

**Affiliations:** 1 Nanjing Medical University, Nanjing, Jiangsu, China; 2 Gusu College of Nanjing Medical University, Nanjing, Jiangsu, China; 3 The Child Healthcare Department of Suzhou Hospital, Nanjing Medical University (Suzhou Municipal Hospital), Suzhou, Jiangsu, China

**Keywords:** constipation, formula, necrotizing enterocolitis, premature infant, sex diff erences

## Abstract

**Background:**

Premature infants (PTI) risk gastrointestinal complications including necrotizing enterocolitis (NEC), impairing catch-up growth. Fecal calprotectin (FC) is an inflammation marker, but its long-term determinants in PTI remain unclear, and the atopic predisposition-FC association is unexplored. This study aimed to identify correlates of FC levels in PTI at 10–18 months corrected age (CA). It was hypothesized that birth weight, history of NEC, formula supplementation, length of neonatal intensive care unit (NICU) hospitalization, constipation, and atopic predisposition are associated with FC levels.

**Methods:**

This cross-sectional study was conducted at a tertiary hospital between June and August 2024. FC was measured using a colloidal gold immunochromatographic assay. Values below the limit of detection were treated as left-censored and were handled by multiple imputation method after natural logarithmic transformation. Multiple linear regression with three parallel sensitivity models was performed to explore factors associated with FC levels.

**Results:**

A total of 109 PTI at 10–18 months CA were enrolled in this cross-sectional study. In fully adjusted models, females showing higher FC levels than males and PTI who received formula supplementation within 6 months CA exhibited lower FC levels than those who received it after 6 months CA. Length of NICU hospitalization showed marginal association in adjusted model but did not survive Bonferroni correction nor sensitivity analyses. Birth weight displayed consistent positive direction across models, albeit with minimal effect size.

**Conclusions:**

Sex and formula supplementation within 6 months CA were correlates of FC levels in PTI at 10–18 months CA. The association with NICU hospitalization appears suggestive and requires further validation.

## Introduction

Premature infants (PTI), born before 37 weeks of gestation, have benefited from improved neonatal intensive care and survival (1, 2). However, immature organ systems predispose PTI to severe infections, growth delays, metabolic disorders, and gastrointestinal complications (1–3). The first postnatal year is a critical window for catch-up growth (4), and such complications may impair survival and developmental catch-up (3, 4).

Fecal calprotectin (FC), a neutrophil-derived protein, is a validated marker of intestinal inflammation (5–7). FC levels surge before and during necrotizing enterocolitis (NEC) episodes (8)and may remain elevated until neonatal intensive care unit (NICU) discharge (9, 10); long-term fluctuations are poorly documented. Existing evidence on FC determinants in PTI largely comes from early postnatal studies. Gestational age-dependent variation in FC levels has been observed (11). Perinatal medical exposures during NICU stay have been negatively associated with FC levels (12, 13), while breast milk exposure has been linked to lower FC values (14). Enteral feeding volume correlates positively with FC levels (15).

Cow's milk protein allergy is associated with elevated FC and gastrointestinal symptoms (16, 17), and is linked to allergic rhinitis, atopic eczema, and respiratory allergies (18, 19). However, no study has investigated the association between atopic predisposition (allergic rhinitis, atopic dermatitis, or respiratory allergy) and FC levels. Most research has been confined to the early postnatal period; data on FC determinants at 10–18 months corrected age (CA) are lacking. This study identified correlates of FC levels in PTI at 10–18 months CA. It was hypothesized that birth weight, history of NEC, formula supplementation, length of neonatal intensive care unit (NICU) hospitalization, constipation, and atopic predisposition are associated with FC levels in this population.

## Methods

### Study design, recruitment, and sample size

This was a cross-sectional observational study conducted at the Pediatric Health Clinic of Suzhou Hospital Affiliated to Nanjing Medical University between June and August 2024.

Sample size estimation: Based on previously reported standard deviations of FC levels and effect sizes of major influencing factors in children (12, 15–17, 20, 21), with a two-sided significance level (*α*) of 0.05, a power (1−*β*) of 0.80, and accounting for an anticipated attrition rate of 15%–20%; the minimum required sample was 120, and 150 participants were initially planned.

Recruitment: Consecutive enrollment (non-probability sampling) was used. During routine pediatric clinics, two trained researchers invited guardians of all eligible PTI. Following explanation of study purpose, procedures, and risks, written informed consent was obtained.

Inclusion criteria (all of the following had to be met): (1) GA < 37 weeks; (2) CA between 10 and 18 months; (3) clinically stable with no signs of acute infection.

Exclusion criteria (meeting any one of the following resulted in exclusion): (1) severe disability, congenital malformations, or chromosomal/genetic defects; (2) maternal oral or intravenous antibiotic use during lactation; (3) maternal mastitis during lactation; (4) history of gastrointestinal surgery; and (5) did not adhere to the World Health Organization (WHO) guidelines for complementary feeding introduction. (6) diarrhea with antibiotic treatment within 1 week prior to stool sampling.

Data collection: Data were collected through face-to-face interviews with the guardians using paper-based questionnaires, and were further complemented and verified by information retrieved from the hospital electronic medical record system. Data cleaning and verification were completed between September and October 2024. Data included: (1) medical record data: date of birth, expected date of delivery, GA (weeks), maternal age (years), birth weight (grams), sex, and length of NICU hospitalization (days). CA was calculated based on the expected date of delivery and recorded in months. (2) Interview/questionnaire data: formula supplementation and breastfeeding cessation within 6 months CA; history of NEC, allergic rhinitis, eczema, wheezing; antibiotic and probiotic use, and gastrointestinal symptoms (diarrhea, constipation) within 1 week before stool collection.

Definitions of key variables: Definitions of key variables were based on parental report via the Infant Related Health Questionnaire (Supplementary File 1). Antibiotic and probiotic use were defined as any oral or intravenous antibiotic administration and any probiotic intake, respectively, within the past week. Diarrhea was defined as a significant increase in stool frequency (≥3 times per day) with watery or pasty consistency within the past week. Constipation was defined as dry, hard stool, difficult defecation, or significantly prolonged defecation interval within the past week. Formula supplementation was defined as having been fed with formula for more than 1 month before 6 months of CA. Breastfeeding cessation was defined as having been breastfed and then having stopped breastfeeding before 6 months of CA. Atopic predisposition was defined as the presence of any of the following: allergic rhinitis (at least one nasal symptom lasting ≥2 consecutive weeks with a cumulative duration of ≥1 h per day), atopic dermatitis (any skin symptom listed in Question 12), or respiratory allergy (wheezing, as defined in Question 13).

### Ethics approval and consent to participate

This research adheres to the ethical principles outlined by the Ethics Committee, Suzhou Municipal Hospital (IRB approval No. K-2022-016-K02). All participants enrolled in this study provided written informed consent, which was obtained from their parents or legal guardians. All procedures involving human participants were conducted in accordance with the ethical principles outlined in the International Ethical Guidelines for Health-related Research Involving Humans issued by the Council for International Organizations of Medical Sciences and the Declaration of Helsinki (1964, revised 2013).

### Sampling and detection methods of FC

Pre-sterilized stool collection tubes were distributed to the participants by trained research personnel. Stool specimens were collected when the infants were between 10 and 18 months CA, consistent with the study inclusion criteria. Guardians were instructed to collect fresh stool specimens (approximately 5 g) from their infants within 1 h of defecation. The specimens were temporarily stored at 2–8 °C and transported to the Department of Clinical Laboratory at Suzhou Hospital Affiliated to Nanjing Medical University within 12 h for testing. Prior to specimen collection, guardians were advised to avoid sampling if the infant had received antibiotics within the preceding week or was in the acute phase of diarrhea.

FC concentrations were measured using the Diagnostic Kit (Colloidal Gold) for Calprotectin (Xiamen Wiz Biotech Co., Ltd.– Xiamen City, Fujian Province, 361026, P.R. China). The manufacturer-provided upper reference limit for healthy adults was 60 μg/g, and the lower limit of detection for the kit was 15 μg/g.

### Data processing

Multiple imputation via Markov Chain Monte Carlo (MCMC) was used as the primary approach for left-censored data (FC concentrations < 15 μg/g). Due to right-skewness, a natural logarithmic transformation [Ln-FC, with ln(FC + 1) for zero values] was performed before imputation. Missing Ln-FC values were imputed using MCMC with five imputations. The imputation model incorporated all candidate variables and auxiliary variables potentially relevant to FC levels or missingness, following the inclusive strategy principle.

### Statistical analysis

Comprehensive data verification and statistical analyses were performed using SPSS version 25.0 (IBM Corp., Armonk, NY, USA) from June to October 2025.

Categorical variables (sex, history of NEC, formula supplementation and breastfeeding cessation within 6 months CA, probiotic use, constipation, atopic predisposition, history of eczema/allergic rhinitis/wheezing) were described as frequencies and percentages; associations with FC levels were examined using the Mann–Whitney U test. Continuous variables (GA, maternal age, birth weight, length of NICU hospitalization, chronological age/CA) with normal distribution were described as mean ± SD, and non-normal as median (IQR); Spearman's rank correlation was used to explore associations with FC levels.

After imputation, univariate analyses were conducted with imputed Ln-FC as the dependent variable. Independent variables were selected for multiple linear regression based on univariate screening (*P* < 0.05) and clinical considerations. Regression estimates from the five imputed datasets were combined using Rubin's rules, and pooled results are presented as final outcomes. Results are reported as unstandardized regression coefficients (B), 95% CI for B, *P*-values, tolerance, and variance inflation factor (VIF).

A *post-hoc* sensitivity analysis was planned using G*Power (version 3.1) to verify whether the achieved sample size was sufficient for the multiple linear regression analysis and to determine the maximum number of independent variables supported by that sample size, in order to avoid model overfitting. Variable selection was based on *P* < 0.05 in univariate analyses combined with clinical expertise. The significance threshold was *P* < 0.05; Bonferroni correction was applied with corrected *α*’ = 0.007.

Model assumptions were tested: (1) multicollinearity: VIF < 10; (2) outliers and influential points: standardized residuals > |3| and Cook's distance > 1; (3) homoscedasticity: Breusch–Pagan test with *P* > 0.05.

To evaluate robustness, three parallel multiple linear regression models were prespecified as sensitivity analyses: Model 1 (basic) included variables with *P* < 0.05 in univariate analyses (sex, formula supplementation, birth weight) and the sex-by-birth-weight interaction term (birth weight centered at its mean). Model 2 (perinatal-hospitalization) additionally adjusted for GA, maternal age, and length of NICU hospitalization. Model 3 (postnatal-functional) additionally adjusted for constipation, atopic predisposition, and CA of PTI.

Sex was coded as 0 for female and 1 for male. Birth weight was centered at its sample mean to reduce structural collinearity and construct the interaction term (product of sex and centered birth weight). Centering does not alter statistical conclusions regarding the interaction.

## Results

### Baseline characteristics of the study population

During recruitment, 133 PTI meeting criteria were enrolled. Exclusions: 11 based on clinical criteria, 3 withdrew consent, 4 had incomplete core questionnaire data, 3 had inadequate stool specimens, and 3 had lost specimens. Ultimately, 109 valid cases were analyzed, yielding an effective enrollment rate of 81.95% (109/133). The study group comprised 60 females (55.0%) and 49 males (45.0%), with mean birth weights of 1,616.07 ± 487.90 g and 1,659.27 ± 452.51 g, respectively (Table 1).

**Table 1:** Baseline characteristics of PTI at 10–18 months CA and univariate analysis of factors associated with FC levels (*N* = 109).

Binary categorical variables	n (%)/M ± SD/P50(P25, P75)	Z/*ρ*	*P*
Sex		−3.443	0.001**^a^
Female	60 (55.0)		
Male	49 (45.0)		
History of NEC		−1.233	0.218^a^
Yes	4 (3.7)		
No	105 (96.3)		
Formula supplementation within 6 months CA		−2.415	0.016*^a^
Yes	98 (89.9)		
No	11 (10.1)		
Breastfeeding cessation within 6 months CA		−1.311	0.190^a^
Yes	67 (61.5)		
No	41 (37.6)		
Probiotic supplementation		−1.363	0.173^a^
Yes	22 (20.2)		
No	87 (79.8)		
Constipation		−0.898	0.369^a^
Yes	13 (11.9)		
No	96 (88.1)		
History of eczema		−1.357	0.175^a^
Yes	24 (22.0)		
No	85 (78.0)		
History of allergic rhinitis		−0.523	0.601^a^
Yes	8 (7.3)		
No	101 (92.7)		
History of wheezing		−1.055	0.291^a^
Yes	14 (12.8)		
No	95 (87.2)		
Atopic predisposition		−1.704	0.088^a^
Yes	39 (36.8)		
No	70 (64.2)		
Maternal age (years)	31.56 ± 3.89	−0.053	0.583^b^
GA (weeks)	32.6 (30.65, 33.90)	0.034	0.723^b^
Birth weight (grams)	1,635.49 ± 470.66	0.227	0.017*^b^
Female	1,616.07 ± 487.90	0.279	0.031*^b^
Male	1,659.27 ± 452.51	0.201	0.167^b^
Length of NICU hospitalization (days)	34.00 (19.00, 53.00)	−0.082	0.395^b^
CA of PTI (months)	12.40 (10.60, 13.80)	0.092	0.339^b^

Table 1 summarizes the sociodemographic and clinical characteristics of the 109 PTI. Categorical variables are presented as frequencies (percentages), and continuous variables are described as mean ± SD or median (IQR) based on normality test results. The final columns show the results of univariate analyses examining the association of each variable with FC levels (Mann–Whitney *U*-test for binary variables and Spearman's rank correlation for continuous variables).

a Mann–Whitney *U*-test.

b Spearman's rank correlation.

* CA: corrected age; FC: fecal calprotectin; GA: gestational age; NEC: necrotizing enterocolitis; NICU: Neonatal Intensive Care Unit; PTI: premature infants.

** *P* ＜ 0.05.

### FC levels

Of the 109 stool samples analyzed, FC levels were non-normally distributed (Shapiro–Wilk, *P* < 0.05), with a median (IQR) of 30.0 (13.5, 46.0) μg/g (range: <15–213 μg/g). 29 cases (26.6% of the total sample) had FC concentrations below the lower limit of detection of the assay kit and were recorded as “< 15 μg/g”.

Outlier diagnostics revealed that standardized residuals ranged from −1.663 to 4.455. Although a few cases had absolute standardized residuals exceeding 3, the maximum Cook's distance was 0.392, well below the conventional threshold of 1.0, indicating the absence of influential cases that could substantially affect the regression coefficient estimates. The maximum center leverage value was 0.211, slightly above the conventional reference value of 0.193; however, in conjunction with the extremely low Cook's distance values, these high-leverage points did not materially perturb the model fit and were therefore retained in the analysis without exclusion. The B–P test yielded a *P*-value > 0.05, suggesting homoscedasticity of residuals and satisfying the assumption of constant variance in linear regression.

### Univariate analysis of factors associated with FC levels

Females showing higher FC levels than males (Z = −3.443, *P* = 0.001). The FC levels in the group with formula supplementation within 6 months CA were significantly higher than those in the group without formula supplementation (Z = −2.415, *P* = 0.016). Birth weight correlated positive correlation with FC levels (*ρ*= 0.227, *P* = 0.017). In sex-stratified analyses, the correlation remained significant in females(*ρ* = 0.279, *P* = 0.031), but not males(*P* = 0.167). No statistically significant associations with FC levels were observed for history of NEC, breastfeeding cessation within 6 months CA, probiotic use, history of eczema/allergic rhinitis/wheezing, atopic predisposition, GA, maternal age, length of NICU hospitalization, chronological age, or CA (all *P* > 0.05) (Table 1).

### Regression model diagnostics and hypothesis testing

Hierarchical multiple linear regression analyses were conducted to construct three progressively adjusted models, with natural log-transformed FC (Ln-FC) as the dependent variable. Independent variables included those with *P* < 0.05 in univariate analyses and clinically relevant confounders. The final effective sample size was 109 cases. *Post-hoc* sensitivity analysis using G*Power (version 3.1) with power 0.80 and *α* 0.05 indicated a minimum detectable effect size (f²) of 0.15, considered medium by Cohen's criteria. Thus, the sample had >80% power to detect medium or larger effects, and a maximum of eight independent variables could be supported. Based on univariate analyses (*P* < 0.05) and clinical expertise, seven independent variables were finally included in the regression model (Table 2). Infants with any history of allergic rhinitis, eczema, or wheezing were classified as having atopic predisposition.

**Table 2:** Hierarchical multivariate linear regression analysis of factors associated with FC levels in PTI at 10–18 months CA (pooled results after multiple imputation, *N* = 109).

Variable		B	95% CI for B	*P*	Tolerance	VIF
Model 1	Intercept	3.360	(2.926, 3.795)	＜0.001***	–	–
	Sex	−0.430	(−0.617, −0.243)	＜0.001***	0.984	1.017
	Formula adding within 6 months CA	0.499	(0.181, 0.817)	0.002**	0.932	1.072
	Birth weight^a^	0.000	(0.000, 0.001)	0.049*	0.583	1.716
	Sex × Birth weight^b^	−0.00° ^c^	(0.000, 0.000)	0.661	0.580	1.724
Model 2	Intercept	3.916	(1.609, 6.223)	0.001**	–	–
	Sex	−0.438	(−0.620, −0.256)	＜0.001***	0.970	1.031
	Formula adding within 6 months CA	0.470	(0.163, 0.778)	0.003**	0.927	1.079
	Birth weight^a^	0.001	(0.000, 0.001)	＜0.001***	0.205	4.879
	Sex × Birth weight^b^	0.000	(−0.001, 0.000)	0.438	0.554	1.806
	Maternal age	−0.020	(−0.044, 0.004)	0.100	0.906	1.103
	GA	−0.034	(−0.103, 0.036)	0.337	0.266	3.761
	Length of NICU hospitalization	0.008	(0.000, 0.015)	0.036*	0.254	3.930
Model 3	Intercept	3.056	(2.373, 3.738)	＜0.001***	–	–
	Sex	−0.392	(−0.581, −0.202)	＜0.001***	0.932	1.072
	Formula adding within 6 months CA	0.507	(0.193, 0.821)	0.002**	0.926	1.080
	Birth weight^a^	0.000	(0.000, 0.001)	0.050	0.546	1.833
	Sex × Birth weight^b^	−0.000^c^	(0.000, 0.000)	0.765	0.542	1.845
	Constipation	0.209	(−0.079, 0.496)	0.154	0.955	1.048
	Atopic predisposition	−0.140	(−0.342, 0.063)	0.174	0.881	1.134
	CA of PTI	0.024	(−0.011, 0.059)	0.176	0.958	1.044

*Model 1: F = 8.689, *P* ＜ 0.001***, Adjusted R^2^ = 0.222; Model 2: F = 6.903, *P* ＜ 0.001***, Adjusted R^2^ = 0.277, Model 3: F = 5.950, *P* ＜ 0.001***, Adjusted R^2^ = 0.243. *B: unstandardized regression coefficients; 95% CI: 95% confidence interval for B; *P*: *P*-values; VIF, variance inflation factor; *CA, corrected age; FC, fecal calprotectin; GA, gestational age; NICU, Neonatal Intensive Care Unit; PTI, premature infants.

a Birth weight centered.

b Interaction based on centered birth weight.

c The coefficient for the sex × birth weight interaction term was −8.947 × 10⁻⁵ (approximately −0.0001), which was not statistically significant (*P* = 0.661, *P* = 0.765, respectively).

* *P* ＜ 0.05.

** *P* ＜ 0.01.

*** *P* ＜ 0.001.

All models demonstrated statistically significant overall fit (all *P* < 0.001), with adjusted R² values ranging from 0.222 to 0.277, indicating that the included covariates accounted for a modest proportion of the variability in FC levels. Collinearity diagnostics showed that all VIFs were below 5 and all tolerance values exceeded 0.1, indicating no significant multicollinearity and supporting the appropriateness of the variable selection.

Model 1 (the basic model) was constructed with sex, formula supplementation within 6 months CA, birth weight, and the sex-by-birth-weight interaction term as independent variables. The model demonstrated statistically significant overall fit (F = 8.689, *P* < 0.001, adjusted R² = 0.222). Significant associations with Ln-FC levels were observed for sex (B = −0.430, 95% CI: −0.617 to −0.243, *P* < 0.001), formula supplementation within 6 months CA (B = 0.499, 95% CI: 0.181–0.817, *P* = 0.002), and birth weight (B = 0.000, 95% CI: 0.000–0.001, *P* = 0.049). The sex-by-birth-weight interaction term did not achieve statistical significance (B = 0.000, 95% CI: 0.000–0.000, *P* = 0.661).

Model 2 (the perinatal-hospitalization model) was constructed by additionally adjusting for maternal age, GA, and length of NICU hospitalization on the basis of Model 1. The model demonstrated statistically significant overall fit (F = 6.903, *P* < 0.001, adjusted R² = 0.277). Significant positive associations with Ln-FC levels were observed for sex (B = −0.438, 95% CI: −0.620 to −0.256, *P* < 0.001), formula supplementation within 6 months CA (B = 0.470, 95% CI: 0.163–0.778, *P* = 0.003), birth weight (B = 0.001, 95% CI: 0.000–0.001, *P* < 0.001), and length of NICU hospitalization (B = 0.008, 95% CI: 0.000–0.015, *P* = 0.036). Maternal age, GA and sex-by-birth-weight interaction term were not statistically significant.

Model 3 (the postnatal-functional model) was constructed by additionally adjusting for constipation, atopic predisposition, and CA of PTI on the basis of Model 1. The model demonstrated statistically significant overall fit (F = 5.950, *P* < 0.001, adjusted R² = 0.243). Significant associations with FC levels were retained for sex (B = −0.392, 95% CI: −0.581 to −0.202, *P* < 0.001) and formula supplementation within 6 months CA (B = 0.507, 95% CI: 0.193–0.821, *P* = 0.002). Birth weight, constipation, atopic predisposition, CA of PTI, and sex-by-birth-weight interaction term were not statistically significant.

In robustness analyses, the sex-by-birth-weight interaction term was not statistically significant across any of the three models with varying covariate adjustment strategies (*P* range: 0.438–0.765), suggesting no significant effect modification by sex on the association between birth weight and FC levels. The main effect of sex remained significant in all models (all *P* < 0.001), with consistent negative coefficients (B < 0), indicating that male PTI had lower FC levels than females at the mean birth weight level. Birth weight was marginally significant in Models 1 and 3 (*P* approached 0.05 in Model 1, and *P* = 0.050 in Model 3), and reached statistical significance in Model 2 (*P* < 0.001), although the effect size was minimal (B = 0.001, corresponding to an approximately 0.1% increase in FC per 1 g increase in birth weight). Given the small magnitude of this effect, its clinical relevance warrants cautious interpretation.

Length of NICU hospitalization was included exclusively in the perinatal-hospitalization model (Model 2), where it demonstrated a positive association with Ln-FC levels (B = 0.008, 95% CI: 0.000–0.015, *P* = 0.036). However, this association did not survive Bonferroni correction for multiple comparisons (*α*’ = 0.007, corresponding to 7 tests). To evaluate the robustness of this result, a series of sensitivity analyses were conducted, including dichotomization by the median, stratification according to the clinically meaningful cut-off of 28 days, and natural logarithmic transformation of the original variable. In all three sensitivity analyses, the association between length of NICU hospitalization and Ln-FC levels failed to reach statistical significance (*P* = 0.119, 0.101, and 0.071, respectively), suggesting limited statistical robustness. Accordingly, this finding should be interpreted as suggestive and requires confirmation in larger cohorts (Supplementary file 2).

## Discussion

This study tested the hypothesis that birth weight, history of NEC, formula supplementation, length of NICU hospitalization, constipation, and atopic predisposition are associated with FC levels in PTI at 10–18 months CA. Among 109 PTI, the median FC concentration was 30.0 (IQR 13.5–46.0) μg/g. In fully adjusted models, females showing higher FC levels than males and PTI who received formula supplementation within 6 months CA exhibited lower FC levels than those who received it after 6 months CA. Length of NICU hospitalization showed a marginal association that did not survive Bonferroni correction or sensitivity analyses. Birth weight displayed a consistent positive direction with minimal effect size. No significant associations were observed for history of NEC, constipation, atopic predisposition, maternal age, CA, or GA. Thus, the hypothesis was only partially supported.

The mean FC concentration in this study was lower than previously reported in PTI during early postnatal life (12, 15, 22). No reference range exists for PTI at this CA. Existing studies mostly examined FC within 6–8 weeks after birth. Given the immature intestinal barrier and immune system of PTI, FC is likely influenced by numerous perinatal and postnatal factors (11, 23).

A 2025 meta-analysis established reference ranges for FC in healthy term infants aged 0–4 years, with 104.70–115.72 μg/g for 10–18-month (4). Our mean FC was considerably lower. Direct comparisons between term and PTI at the same CA are scarce. Few studies comparing at birth consistently reported higher FC in PTI (12, 15, 24). Further research on longitudinal FC trajectories in PTI is needed.

We found no significant association between CA and FC, inconsistent with age-related decline reported in healthy children (20, 23). This may reflect differences in the study population. Most reference data are derive from healthy term infants, whereas our study population consisted of PTI, in whom FC levels exhibit a more intricate pattern of change (11). Wide inter- and intra-individual variability in FC levels has been reported in preterm infants during the early postnatal period (12, 15, 25).

FC was measured by colloidal gold immunochromatographic assay (GICA). Although enzyme-linked immunosorbent assay (ELISA) is the reference standard, GICA offers practical advantages for outpatient settings. Comparative studies indicate that GICA exhibit satisfactory concordance with ELISA in both qualitative and semi-quantitative FC assessments, with comparable coefficient of variation and high correlation (r = 0.945 and 0.942) (26). Methodological discrepancies mainly affect absolute concentrations, not relative differences. Since our primary objective was between-group comparison, this bias is unlikely to have affected findings. Moreover, geographic and racial or ethnic variations may also represent potential sources of variability in FC measurements (7, 12, 27).

Higher FC levels were observed in female PTI than in male PTI, consistent with previous findings. Paysal et al., in a cohort of 157 PTI, found higher FC in female neonates (300.5 vs. 110.5 μg/g, *P* < 0.001) (28). The mechanisms are likely multifaceted. From the perspective of sex hormone regulation of the immune system, preterm infants are particularly sensitive to the immunomodulatory effects of sex hormones. McGovern et al. showed that the inhibitory effect of estradiol on IL-1β expression was most pronounced in PTI, and that female PTI exhibited greater responsiveness to the anti-inflammatory effects of progesterone than male PTI (29). However, the effects of sex hormones on neutrophil function may differ from their inhibitory effects on pro-inflammatory cytokines. As a neutrophil-derived protein, FC levels depend on neutrophil migration into the intestinal lumen and degranulation, rather than directly reflecting cytokine concentrations (30). Therefore, higher FC levels in female PTI may reflect sex differences in neutrophil-associated intestinal immune activation, rather than differences in pro-inflammatory cytokine levels. Sex hormone regulation of neutrophil chemotaxis, adhesion, and degranulation, as well as the impact of sex differences in gut microbiota on the mucosal immune microenvironment, may be potential mechanisms underlying this phenomenon. These speculations require validation through simultaneous assessment of neutrophil function, gut microbiota metagenomics, and sex hormone levels.

In univariate analyses, birth weight correlated positive correlation with FC levels. In sex-stratified analyses, the correlation remained significant in females, but not males. After inclusion of a sex-by-birth-weight interaction term and adjustment for other covariates, the interaction term was not statistically significant across any of the three models. Univariate correlation analysis did not account for potential confounders; the significant positive correlation between birth weight and FC levels in female PTI may have been confounded by other birth weight-related perinatal variables, including GA and duration of hospitalization. After adjustment for these covariates, the apparent sex-specific effect was no longer evident. In Models 1 and 3, *P*-values for birth weight approached the conventional 0.05 threshold, indicating marginal statistical significance. In Model 2, inclusion of perinatal covariates closely associated with birth weight, specifically GA and length of NICU hospitalization, yielded a *P*-value of birth weight below 0.001. However, the magnitude of the effect remained modest (approximately a 0.1% increase in Ln-FC per 1 g increment in birth weight). The clinical relevance of birth weight as a correlate of FC levels in PTI at 10–18 months CA warrants cautious interpretation. Future investigations with expanded sample sizes are needed to confirm this interaction pattern.

In univariate analysis, PTI without formula supplementation within 6 months CA exhibited lower FC levels. After adjustment, absence of formula supplementation was associated with higher FC. The discordant direction warrants careful clinical and statistical consideration. In clinical practice, exclusively breastfed PTI tended to have less severe baseline conditions, shorter NICU hospitalization, and greater GA. In univariate analysis, this healthier subgroup had lower FC, creating a spurious protective signal. After adjusting for confounders, the two groups were compared under equivalent disease severity. The association between formula supplementation and FC might more closely approximate the true association, or at least reflect mechanisms beyond residual confounding.

This study found PTI exclusively breastfed within 6 months CA had higher FC levels at 10–18 months CA than those who received formula supplementation. Although this finding seemingly contradicts the conventional notion that breast milk is anti-inflammatory, it does not. It more likely reflects breast milk-driven intestinal immune maturation and host defense activation rather than aggravated pathological inflammation. Groer et al. reported that FC levels increased over time in exclusively breastfed very low birth weight infants but decreased in mixed-fed infants. Neutrophils, monocytes, and chemokines such as IL-8 in breast milk may drive this response (31). A study on human milk fortifier also noted: “Healthy exclusively breastfed infants have higher FC levels than formula-fed infants; elevated FC may not always represent pathological inflammation in PTI, but rather normal maturation of feeding patterns and/or the intestinal immune axis” (32). Willers et al. further demonstrated that S100A8/A9 (constituent proteins of calprotectin) are abundant in breast milk and promote regulatory T cell development and inhibit excessive proliferation of intestinal pathogens in mouse models (33). Baldassarre et al. proposed that physiological elevation of FC in early infancy may represent physiological activation of host defense mechanisms against pathogens rather than a mere inflammatory marker (34).

Although formula provides exogenous nucleotides and modulating microbiota composition (35), substantial high-quality evidence indicates that human milk is the optimal nutrition for preterm infants and confers well-established intestinal protection. It inhibits TLR4 signaling, strengthens the epithelial barrier, promotes healthy gut microbiota establishment, and provides secretory immunoglobulin A, lactoferrin, lysozyme, human milk oligosaccharides, growth factors, and other bioactive components (36–39). Multiple systematic reviews and meta-analyses have consistently confirmed that formula feeding significantly increases the risk of NEC in PTI (40–42). Therefore, breastfeeding should remain firmly prioritized in clinical decision-making for preterm infants.

It should be emphasized that this cross-sectional study did not record detailed information on formula type, timing, amount, duration, or breastfeeding intensity. Moreover, PTI requiring formula supplementation within 6 months CA may have differed from exclusively breastfed PTI in baseline characteristics, feeding intolerance, or maternal milk insufficiency. Thus, the observed associations may be confounded.

In the perinatal-hospitalization model (Model 2), length of NICU hospitalization was positively associated with FC. Nevertheless, this did not survive sensitivity analyses, indicating limited robustness. Length of NICU hospitalization is a multifactorial composite measure that encompasses disease severity and treatment intensity et al. Its relationship with FC may be susceptible to residual confounding. The length of NICU hospitalization in our research was heavily right-skewed (median: 34 days; range: 6–111 days), and the observed positive correlation may have been disproportionately influenced by a few infants with extended hospitalizations. Evidence directly linking NICU stay to FC is scarce.

Evidence directly linking the length of NICU hospitalization to FC levels is scarce in the current literature. Accumulating data indicate that multiple factors encountered during NICU hospitalization may contribute to FC variability. A longitudinal study of 49 PTI, with twice-weekly stool sampling over the first 4 weeks of NICU admission, documented substantial inter- and intra-individual variation in FC levels (mean 268.7 ± 261.3 μg/g), and identified significant associations between elevated FC levels and cumulative pain or stress and illness severity scores (12), which were corroborated by a 2022 study (13). Nevertheless, both of the above-mentioned studies were restricted to the early postnatal phase (4–6 weeks after birth) in PTI, and to date, no study has examined the medium- to longer-term effects of length of NICU hospitalization on FC levels in PTI, such as those assessed at 10–18 months CA (12, 13).

Future investigations with expanded sample sizes and detailed documentation of clinical exposures, including the frequency of painful procedures, and time to achieve full enteral feeding, are needed to further elucidate this association.

Previous studies suggest that children with allergic enteritis and cow's milk protein allergy tend to have higher FC levels (43). In this research, the participants predominantly had mild eczema and allergic rhinitis. They were young and did not display clear signs of food intolerance.

This negative finding is consistent with Baldassarre et al., who followed 109 newborns up to 2 years and found that FC had no predictive value for allergic diseases (44). They proposed that elevated FC in the neonatal period may reflect host defense rather than pathological inflammation, and that allergic diseases are associated with intestinal barrier immaturity and microbiota development (44). In this study, the allergic phenotype mainly consisted of mild skin and respiratory symptoms, without gastrointestinal allergic manifestations (44), which may partly explain this negative result. Future studies may stratify by allergy severity or include older children to further investigate this potential association.

### Limitations

The present study was designed as a cross-sectional observational investigation, which can only reveal associations between the factors of interest and FC levels, and cannot establish the temporal sequence of causality between sex, formula supplementation within 6 months CA, birth weight, length of hospital hospitalization, and FC levels.

Fecal samples were collected at a single time point (10–18 months CA) for FC measurement, and longitudinal FC trajectories were not available for individual infants. As a sensitive biomarker of intestinal inflammation, FC levels in early infancy are subject to substantial fluctuations due to various factors. Consequently, the present study could not assess intra-individual changes in FC levels from the neonatal period to infancy, nor could it distinguish between persistent and transient inflammation in terms of their effects on FC levels. Future studies adopting longitudinal cohort designs with repeated FC measurements at multiple prespecified time points (e.g., 3, 6, 12, and 18 months CA), combined with dietary records and documentation of infectious events, are warranted to more comprehensively elucidate the temporal causal relationships among feeding practices, intestinal inflammation, and dynamic changes in FC levels.

Owing to the cross-sectional design and sample size constraints, we were unable to obtain data on several potentially important confounders, including detailed daily milk intake, formula type, recent use of medications (e.g., proton pump inhibitors, H2-receptor antagonists), Apgar score at birth, requirement for respiratory support, as well as maternal dietary habits and atopic history. The omission of these variables may give rise to residual confounding, which could compromise the precision of our effect estimates.

NEC constitutes one of the most critical intestinal emergencies in PTI. Accumulating evidence has demonstrated a marked elevation in FC levels during the acute phase of NEC, with systematic reviews and meta-analyses reporting diagnostic sensitivities ranging from 0.86 to 0.91 and specificities from 0.79 to 0.93 (8, 45). Nevertheless, the existing evidence is exclusively confined to the acute stage, and no studies have addressed the long-term effects of a history of NEC on FC levels in PTI at later CA, such as 10–18 months.

In the current study, the four infants with a history of NEC all presented with mild neonatal disease (modified Bell stage IIa) (46), and the extremely limited sample size precluded any meaningful statistical inference. Although it has been suggested that NEC survivors are at increased risk of parenteral nutrition-associated cholestasis, short bowel syndrome, neurodevelopmental impairment, and growth retardation (47), whether NEC confers a sustained impact on intestinal inflammatory status remains to be determined.

Given the extreme sample size constraint (*n* = 4), the variable of NEC was excluded from the final multiple regression model, a methodological decision that has been explicitly stated in the Methods section. Future multicenter investigations with larger cohorts of NEC survivors and prospective longitudinal designs are warranted to elucidate the potential long-term influence of NEC on intestinal inflammation and its clinical relevance.

Nevertheless, length of NICU hospitalization was incorporated into our regression models as a composite proxy indicator for overall disease severity. In the context of neonatal intensive care, infants with low Apgar scores or those needing respiratory support almost invariably have prolonged hospitalizations. Thus, length of NICU hospitalization likely accounted for a substantial portion of the variance attributable to disease severity at birth, thereby limiting the potential residual influence of unmeasured confounders on our primary estimates.

## Conclusion

Sex appeared to be a correlate of FC levels in PTI at 10–18 months CA, with males exhibiting lower FC levels than females at the mean birth weight level, independent of birth weight. However, causality cannot be inferred from these data. Formula supplementation within 6 months CA showed a negative association with elevated FC levels, suggesting that it may represent a factor associated with FC levels in this population, although the causal direction warrants further prospective investigation. The association between length of NICU hospitalization and FC levels showed limited statistical robustness and should be regarded as suggestive, requiring confirmation in larger samples. Birth weight might also be a correlate of FC levels, albeit with a modest effect size (approximately a 0.1% increment in FC per 1 g increase), and its clinical relevance should be interpreted cautiously. No statistically significant associations with FC levels were observed for constipation, maternal age, atopic predisposition, CA of PTI, or GA in the adjusted model.

## Data Availability

The raw data supporting the conclusions of this article will be made available by the authors, without undue reservation.
