# Supplementary material for: Sex and formula supplementation are correlates of fecal calprotectin in premature infants at 10–18 months corrected age
Source: Front Pediatr. 2026 Oct 14;14:1743276. doi: 10.3389/fped.2026.1743276 (PMC13713833; doi:10.3389/fped.2026.1743276)
Supplement: Supplementary file 1 [file Supplementaryfile2.docx]

**Supplementary file 2**

**Supplementary Table S1.** Multivariate linear regression analysis of FC levels using LOD/2 substitution method (sensitivity analysis).

| **FC content** | | **B** | **95% CI** | ***P*** | **Tolerance** | **VIF** |
| --- | --- | --- | --- | --- | --- | --- |
| Model 1 | Intercept | 30.974 | (23.430, 38.518) | ＜0.001*** |  |  |
|  | Formula adding after 6 months CA | 32.999 | (10.635, 55.363) | 0.004** | 0.999 | 1.001 |
|  | Constipation | 28.795 | (8.010, 49.581) | 0.007** | 0.999 | 1.001 |
| Model 2 | Intercept | 35.112 | (-21.253, 91.477) | 0.220 |  |  |
|  | Formula adding after 6 months CA | 33.044 | (10.565, 55.523) | 0.004** | 0.998 | 1.002 |
|  | Constipation | 28.611 | (7.579, 49.643) | 0.008** | 0.985 | 1.015 |
|  | Maternal age | -0.131 | (-1.893, 1.632) | 0.883 | 0.985 | 1.015 |
| Model 3 | Intercept | -27.038 | (-197.691, 143.616) | 0.754 |  |  |
|  | Formula adding after 6 months CA | 33.558 | (10.581, 56.535) | 0.005** | 0.963 | 1.038 |
|  | Constipation | 26.231 | (4.775, 47.687) | 0.017* | 0.954 | 1.048 |
|  | Maternal age | -0.254 | (-2.057, 1.549) | 0.781 | 0.949 | 1.054 |
|  | Length of NICU hospitalization | 0.113 | (-0.375, 0.601) | 0.648 | 0.322 | 3.107 |
|  | Atopic predisposition | -8.197 | (-22.663, 6.269) | 0.264 | 0.96 | 1.042 |
|  | Gestational age | 2.050 | (-2.806, 6.906) | 0.404 | 0.314 | 3.19 |
|  | History of NEC | -17.836 | (-55.845, 20.173) | 0.354 | 0.904 | 1.107 |
| Model 4 | Intercept | -35.861 | (-205.344, 133.623) | 0.676 |  |  |
|  | Formula adding after 6 months CA | 32.177 | (9.407, 54.947) | 0.006** | 0.979 | 1.021 |
|  | Constipation | 26.974 | (5.594, 48.355) | 0.014* | 0.959 | 1.042 |
|  | Maternal age | -0.330 | (-2.125, 1.464) | 0.716 | 0.956 | 1.046 |
|  | Length of NICU hospitalization | 0.108 | (-0.380, 0.596) | 0.661 | 0.322 | 3.106 |
|  | Atopic predisposition | -7.875 | (-22.313, 6.563) | 0.282 | 0.962 | 1.040 |
|  | Gestational age | 2.385 | (-2.415, 7.185) | 0.327 | 0.320 | 3.121 |

**P*＜0.05*, *P*＜0.01**, *P*＜0.001***.

*Model 1: F = 7.822, *P* = 0.001**, Adjusted R^2^ = 0.112; Model 2: F = 5.174, *P* = 0.002**, Adjusted R^2^ = 0.104, Model 3: F = 2.667, *P* = 0.014*, Adjusted R^2^ = 0.098, Model 4: F = 2.971, *P* = 0.010*, Adjusted R^2^ = 0.099.

*B: unstandardized regression coefficients; 95% CI: 95% confidence interval for B; P: P-values; VIF: variance inflation factor.

*NEC: necrotizing enterocolitis; FC: fecal calprotectin; CA: corrected age; NICU: Neonatal Intensive Care Unit.

Hierarchical multiple linear regression with stepwise adjustment was performed. Model 1 was unadjusted, and Model 4 was fully adjusted (excluding NEC due to n = 4).

Formula supplementation within 6 months CA was consistently associated with higher FC across all models, remaining significant after Bonferroni correction (α' = 0.007) (Model 1: B = 33.00, *P* = 0.004; Model 4: B = 32.18, *P* = 0.006). Constipation also showed positive associations (Model 1: B = 28.80, *P* = 0.007; Model 4: B = 26.97, *P* = 0.014), but the *P*-value in the fully adjusted model slightly exceeded the corrected threshold, suggesting a suggestive association. Maternal age, NICU stay, atopy, and gestational age were not significant (all *P* > 0.05).

All four models demonstrated a statistically significant overall fit; however, the adjusted R² values were low (0.099–0.112), indicating limited explanatory power. VIF values were < 3.2 and tolerance > 0.1, indicating no multicollinearity.

**Table 2.** Hierarchical multivariate linear regression analysis of factors influencing FC levels (pooled results after multiple imputation).

| **Variable** | | **B** | **95% CI for B** | ***P*** | **Tolerance** | **VIF** |
| --- | --- | --- | --- | --- | --- | --- |
| Model S1 | Intercept | 5.742 | (3.393, 8.091) | ＜0.001*** | 3.393 | 8.091 |
|  | Sex | -0.443 | (-0.627, -0.260) | ＜0.001*** | -0.627 | -0.260 |
|  | Formula adding within 6 months CA | 0.46 | (0.150, 0.771) | 0.004** | 0.150 | 0.771 |
|  | Birth weight ^a^ | 0.001 | (0.000, 0.001) | 0.001** | 0.000 | 0.001 |
|  | Sex * Birth weight ^b^ | -0.000^c^ | (0.000, 0.000) | 0.623 | 0.000 | 0.000 |
|  | Maternal age | -0.015 | (-0.039, 0.009) | 0.208 | -0.039 | 0.009 |
|  | GA | -0.055 | (-0.119, 0.009) | 0.092 | -0.119 | 0.009 |
|  | Length of NICU hospitalization | 0.193 | (-0.050, 0.436) | 0.119 | -0.050 | 0.436 |
| Model S2 | Intercept | 5.626 | (3.241, 8.011) | ＜0.001*** | 3.241 | 8.011 |
|  | Sex | -0.465 | (-0.648, -0.281) | ＜0.001*** | -0.648 | -0.281 |
|  | Formula adding within 6 months CA | 0.466 | (0.156, 0.776) | 0.004** | 0.156 | 0.776 |
|  | Birth weight ^a^ | 0.001 | (0.000, 0.001) | 0.001** | 0.000 | 0.001 |
|  | Sex * Birth weight ^b^ | 0.000 | (-0.001, 0.000) | 0.566 | -0.001 | 0.000 |
|  | Maternal age | -0.014 | (-0.038, 0.010) | 0.266 | -0.038 | 0.010 |
|  | GA | -0.054 | (-0.118, 0.010) | 0.099 | -0.118 | 0.010 |
|  | Length of NICU hospitalization | 0.203 | (-0.040, 0.446) | 0.101 | -0.040 | 0.446 |
| Model S3 | Intercept | 5.067 | (2.424, 7.709) | ＜0.001*** | 2.424 | 7.709 |
|  | Sex | -0.472 | (-0.656, -0.288) | ＜0.001*** | -0.656 | -0.288 |
|  | Formula adding within 6 months CA | 0.480 | (0.171, 0.790) | 0.003** | 0.171 | 0.790 |
|  | Birth weight ^a^ | 0.001 | (0.000, 0.001) | 0.001** | 0.000 | 0.001 |
|  | Sex * Birth weight ^b^ | 0.000 | (-0.001, 0.000) | 0.377 | -0.001 | 0.000 |
|  | Maternal age | -0.018 | (-0.042, 0.006) | 0.148 | -0.042 | 0.006 |
|  | GA | -0.049 | (-0.114, 0.016) | 0.142 | -0.114 | 0.016 |
|  | Length of NICU hospitalization | 0.246 | (-0.022, 0.513) | 0.071 | -0.022 | 0.513 |

**P*＜0.05*, *P*＜0.01**, *P*＜0.001***.

*Model S1: F = 6.494, *P* ＜ 0.001***, Adjusted R^2^ = 0.263; Model S2: F = 6.547, *P* ＜ 0.001***, Adjusted R^2^ = 0.264, Model S3: F = 6.667, *P* ＜ 0.001***, Adjusted R^2^ = 0.269.

*B: unstandardized regression coefficients; 95% CI: 95% confidence interval for B; *P*: *P*-values; VIF: variance inflation factor.

*CA: corrected age; FC: fecal calprotectin; GA: gestational age; NICU: Neonatal Intensive Care Unit; PTI: preterm infants.

^a^ birth weight centered.

^b^ interaction based on centered birth weight.

c The coefficient for the sex × birth weight interaction term was -9.92 × 10⁻⁵ (approximately -0.0001), which was not statistically significant (P = 0.623).

Notes: All models were adjusted for sex, formula supplementation by 6 months CA, birth weight (centered), maternal age, GA, and the sex * birth weight interaction term. S1: length of NICU hospitalization dichotomized by the median (≤34 vs. >34 days); S2: dichotomized by the clinically relevant cut-off of 28 days (≤28 vs. >28 days); S3: natural logarithmic transformation of length of NICU hospitalization. P values for the length of NICU hospitalization variable were 0.119, 0.101, and 0.071, respectively, none reaching statistical significance. All VIF values were < 5, indicating no significant multicollinearity.
