# Supplementary material for: Sex and formula supplementation are correlates of fecal calprotectin in premature infants at 10–18 months corrected age
Source: Front Pediatr. 2026 Oct 14;14:1743276. doi: 10.3389/fped.2026.1743276 (PMC13713833; doi:10.3389/fped.2026.1743276)
Supplement: Supplementary file 2 [file Supplementaryfile1.doc]

**Infant Related Health Questionnaire**

**Serial No.**: ________ **ID No.**: ________ **Name**: ________ **Gender**: ________ **Age**: ________ **Phone No.**: ________

Please answer the following questions in accordance with your baby’s actual situation.

1. **Has your baby taken antibiotics orally or intravenously in the past week?**

□ Yes □ No

2. **Has your baby taken probiotics in the past week?**

□ Yes (Please fill in the probiotic brand: ________________) □ No

3. **Has your baby had diarrhea in the past week? What is the stool consistency? (Significant increase in stool frequency ≥ 3 times a day, watery/pasty stool)**

□ Yes (Watery / Pasty) □ No

4. **Has your baby had constipation in the past week? (Dry, hard stool, difficult defecation or significant prolongation of defecation interval)**

□ Yes □ No

5. **Has your baby had other intestinal problems recently? (e.g., blood in stool, vomiting, blood in vomit)**

□ Yes (Please describe specific symptoms: ________________) □ No

6. **Has your baby ever been fed with formula milk for more than 1 month before 6 months of corrected age (Corrected age: calculated from due date)?**

□ Yes □ No

7. **Has your baby ever been breastfed and then stopped breastfeeding before 6 months of corrected age (Corrected age: calculated from due date)?**

□ Yes □ No

8. **Age at start and stop of breastfeeding (Chronological age: calculated from the date of birth)**

Start age: ________ months Stop age: ________ months

**9. Please tick √ for the types of complementary food your baby is currently having, If no complementary foods have been introduced yet, write “None”.**

□ Rice cereal □ Staple foods □ Vegetables □ Fruits □ Egg white □ Egg yolk □ Poultry □ Fish and shrimp □ Pulses

10. **Please tick √ if your baby has had any of the following symptoms (lasted for at least 2 consecutive weeks with a cumulative duration of ≥ 1 hour per day)**

□ Rubbing/picking/rubbing the nose (Tick if at least one applies)

□ Sneezing

□ Watery nasal discharge

□ Nasal congestion

11. **Please tick √ if your baby has ever had any of the following skin symptoms**

**□** Frequent scratching of the skin

□ Dry skin

□ Skin redness (patchy redness, similar to allergy or sunburn)

□ Dense small bumps/rashes on the skin (rough and raised to the touch)

□ Transparent or pale-yellow fluid oozing from broken skin (wet surface)

□ Thickened, hardened skin with deepened lines at the affected area (rough to the touch, like tree bark)

12. **Has your baby ever had wheezing?**

□ Yes (Please tick √ for any applicable situation below)

□ A high-pitched hissing/squeaking/roaring sound when breathing

□ Doctor detected wheezing/rhonchi in the lungs during auscultation

□ No

Notes: Final enrollment was contingent upon the successful introduction of all complementary food types enumerated in Question 9.
